# Glutathione: new roles in redox signaling for an old antioxidant

**DOI:** 10.3389/fphar.2014.00196

**Published:** 2014-08-26

**Authors:** Katia Aquilano, Sara Baldelli, Maria R. Ciriolo

**Affiliations:** ^1^Department of Biology, University of Rome Tor VergataRome, Italy; ^2^Scientific Institute for Research, Hospitalization and Health Care, Università Telematica San Raffaele RomaRome, Italy

**Keywords:** nitric oxide, viral infection, autophagy, redox signal, reactive oxygen species

## Abstract

The physiological roles played by the tripeptide glutathione have greatly advanced over the past decades superimposing the research on free radicals, oxidative stress and, more recently, redox signaling. In particular, GSH is involved in nutrient metabolism, antioxidant defense, and regulation of cellular metabolic functions ranging from gene expression, DNA and protein synthesis to signal transduction, cell proliferation and apoptosis. This review will be focused on the role of GSH in cell signaling by analysing the more recent advancements about its capability to modulate nitroxidative stress, autophagy, and viral infection.

## INTRODUCTION

γ-L-glutamyl-L-cysteinyl-glycine chiefly known as glutathione (GSH) is required for several cell processes interconnected with alterations in the maintenance and regulation of the thiol-redox status, due to its capability to exist in different redox specie (Forman et al., 2009). Under physiological conditions the reduced GSH is the major form with its concentration from 10 to 100-folds higher than the oxidized species (oxidized GSH, GSSG and mixed disulphide, GSSR). GSSG is predominantly produced by the catalysis of GSH peroxidase (GPX) as well as from the direct reactions of GSH with electrophilic compounds, e.g., radical species. The production of GSSR, instead, requires a “reactive” cysteinyl residue, which at physiological pH is present in the thiolate form. These residues, under oxidative stress, are prone to oxidation in sulfenic acid, which efficiently reacts with GSH leading to a glutathionylated-cysteine derivative (GSSR). Both GSSG and GSSR can be catalytically reduced back to GSH by the NADPH-dependent GSH reductase and thioredoxin (Trx)/glutaredoxin (Grx) system, respectively. Additionally, the non-enzymatic inter-conversion between GSSG and GSSR can occur. Therefore, the ratio between reduced and oxidized forms of GSH is an important indicator of the redox environment and, at the same time, contributes to the accomplishment of the molecular mechanisms underlying cell proliferation, differentiation or death in the form of apoptosis (Dickinson and Forman, 2002). Moreover, it is now well established that the reversible formation of mixed disulfide GSSR through protein-*S*-glutathionylation is an important on/off mechanism for dynamic post-translational regulation of a variety of regulatory, structural and metabolic proteins involved in signaling and metabolic pathway in cell systems (Ghezzi, 2005, 2013). In this context, we also demonstrated that the disruption of the cellular redox buffer, controlled by GSH, increases not only the oxidative stress (Filomeni et al., 2005a), but also the endogenous physiological flux of NO in neuronal cells (Aquilano et al., 2011a, 2013). Indeed, GSH decrease causes protein nitration, *S*-nitrosylation, and DNA strand breaks in neuronal cells. Such alterations were also associated with inhibition of cytochrome *c* oxidase (CcOX) activity and microtubule network disassembly, which are considered hallmarks of nitric oxide (NO) toxicity, indicating that NO, rather than the depletion of GSH per se, is the primary mediator of cell damage. These results support the hypothesis that GSH represents the most important buffer of NO toxicity in neuronal cells. Moreover, the same NO/cGMP signaling was effective in survival cell response to GSH depletion in skeletal muscle cells (Aquilano et al., 2013).

On the basis of these knowledge, it is not surprising that alterations in GSH homeostasis have been implicated in the etiology and/or progression of many human diseases (Ballatori et al., 2009). In fact, a decrement of GSH levels contributes to oxidative stress associated with aging and many pathological states, including neurodegeneration, inflammation, and infections. However, given the many roles played by GSH, it has been difficult to ascribe causal relationships between changes in GSH levels or redox state and development of disease (Liu et al., 2004).

In this review we briefly summarize the mechanisms of GSH synthesis and homeostasis, while we will focus on the strict connection between GSH levels and oxidative/nitrosative stress and on the downstream redox changes that modulate signaling pathways involved in viral infection and autophagy (ATG).

## GSH HOMEOSTASIS AND SYNTHESIS

Eukaryotic cells have distinct reservoirs of GSH: the majority of GSH (almost 90%) is in the cytosol, which also represents the main place for its synthesis; from cytosol, GSH is distributed into organelles such as mitochondria, nucleus and endoplasmic reticulum (Forman et al., 2009; Lu, 2013). In these districts GSH is predominantly in the reduced form except for endoplasmic reticulum where the oxidized form is mainly present being necessary for the correct folding and secretory pathway of proteins (Hwang et al., 1992).

GSH is the most important hydrophilic antioxidant that protects cells against exogenous and endogenous toxins, including reactive oxygen (ROS) and nitrogen (RNS) species (Rauhala et al., 2005; Jozefczak et al., 2012). Among such species the radical forms are removed *via* non-enzymatic reduction with GSH, whereas the elimination of hydroperoxides requires enzymatic catalysis by GPX and catalase. The resulting oxidized form of GSH (GSSG), characterized by a disulfide bond between two molecules of GSH is efficiently reduced back to GSH by the NADPH-dependent catalysis of the flavoenzyme GSH reductase. Indeed, the GSH and GSH-related enzymatic systems are efficient tools that cells have exploited in detoxification and, at the same time, represent the most ancient notice on the physiological role played by the tripeptide. GSH in fact is both a nucleophile and a reductant, and therefore can react with electrophilic or oxidizing species rendering the former molecules more soluble and excretable, and the latter unable to interact with more critical cellular constituents such as lipids, nucleic acids and proteins. Conjugation of GSH with electrophilic compounds is mainly mediated by the glutathione-*S*-transferases (GSTs), a super family of Phase II detoxification enzymes.

GSH is synthesized *in vivo,* by the consecutive action of two ATP-dependent enzymes, from the precursor amino acids cysteine, glutamate and glycine. The first enzyme, glutamate–cysteine ligase (GCL) formerly called γ-glutamylcysteine synthase (GCS) is the rate-limiting enzyme. GCL is a heterodimer that can be dissociated under non-denaturing conditions into a modulatory or light subunit (GCLM), and a catalytic or heavy subunit (GCLC; Lu, 2013). GCL forms an unusual peptide bond between the γ-carboxyl of glutamate and the amino group of cysteine using the energy provided by the hydrolysis of ATP (Lu, 2013). GCL belongs to the class of proteins that are sensitive to oxidative stress, and its expression is mainly under the regulation of the Nuclear factor (erythroid-derived 2)-like 2 (NFE2L2), a transcription factor that regulates a wide array of antioxidant responsive element-driven genes in various cell types (Baldelli et al., 2013). The second enzyme required for *the novo* GSH biosynthesis is glutathione synthase (GS). The human GS enzyme is a homodimer of subunits containing 474 amino acid residues, encoded by a single-copy gene (Gali and Board, 1997). It catalyzes the addition of glycine to γ-glutamylcysteine created by GCL to form GSH, γ-L-glutamyl-L-cysteinyl-glycine, a reaction again driven by the hydrolysis of ATP. Finally, the chemical structure of GSH provides peculiar characteristics ranging from un-susceptibility to proteolysis to redox thiols catalysis. The overall rate of GSH synthesis is controlled by several factors including: (i) availability of the substrate mainly L-cysteine (Anderson and Meister, 1983); (ii) amount and relative ratio between the two subunits of GCL (Chen et al., 2005); (iii) extent of feedback inhibition of GCL by GSH (Taylor et al., 1996). Additionally, in some cases, the provision of ATP for GSH synthesis could represent another limiting factor. Even if virtually, all cell types synthesize GSH, the main source of the tripeptide is liver where the bulk of cysteine, the rate limiting amino acid, derived from diet is metabolized. After its synthesis, GSH is delivered to some intracellular compartments, including mitochondria, endoplasmic reticulum, nucleus, and to the extracellular space (e.g., blood plasma and bile) for utilization by other cells and tissues (Forman et al., 2009).

In contrast to GSH synthesis, which occurs intracellularly, GSH degradation occurs exclusively in the extracellular space, and in particular, on the surface of cells that express the enzyme γ-glutamyl transpeptidase (also called γ-glutamyl transferase, GGT; Ballatori et al., 2009; Baudouin-Cornu et al., 2012). The GGT is the only enzyme that can initiate catabolism of GSH and GSH-adducts (e.g., GSSG, glutathione *S*-conjugates, and glutathione complexes). GGT is an heterodimeric glycoprotein located on the external plasma membrane of specific cells present in kidney tubules, biliary epithelium and brain capillaries where it hydrolyses GSH into glutamic acid and cysteinyl-glycine; this dipeptide is further hydrolysed by cell surface dipeptidases and the resulting amino acids taken up by cells for regeneration of intracellular GSH (Pompella et al., 2007). The intra- and extracellular GSH levels are determined by the balance between its production, consumption, and transportation. Due to important physiological functions of GSH, these processes are tightly regulated. The activities of the enzymes involved in GSH metabolism are controlled at transcriptional, translational, and post-translational levels.

## GSH AND OXIDATIVE STRESS

The investigation on the field related to production of ROS/RNS during metabolic processes, even under physiological conditions, unlocks another branch of research focusing on the role of GSH ranging from antioxidant/radical scavenger to redox signaling modulator. GSH effectively scavenges free radicals and other ROS and RNS (e.g., hydroxyl radical, lipid peroxyl radical, superoxide anion, and hydrogen peroxide) directly and indirectly through enzymatic reactions. The chemical structure of GSH determines its functions, and its broad distribution among all living organisms reflects its important biological role (Johnson et al., 2012). In particular, it has long been established that the thiol moiety of GSH is important in its antioxidant function in the direct scavenge of radical species. Indeed, the one-electron reduction with radicals is not chemically favorable, because it would generate the unstable thiyl radical GS. However, the reaction is kinetically driven in the forward direction by the removal of GS through the following reactions with thiolate anion (GS^-^) and then with oxygen. The first reaction leads to the generation of GSSG^-^, which in the presence of O_2_, generates GSSG, and superoxide ($O_{2}^{•-}$). Ultimately, the radical chain reactions will be blocked by the antioxidant enzymes superoxide dismutase (SOD) in association with catalase or GPX that determines the complete free radicals scavenging (Winterbourn, 1993). Indeed, we demonstrated that GSH finely compensates the decline of SOD1 activity in: (i) cells expressing less active SOD1 mutant found in familial amyotrophic lateral sclerosis (Ciriolo et al., 2001); (ii) cells in which SOD1 is down-regulated by RNA interference (Aquilano et al., 2006; Vigilanza et al., 2008). In particular, the initial burst of superoxide that cannot be eliminated efficiently due to the inactivity of SOD1 is promptly buffered by the induction of GSH synthesis resulting in protection against oxidative stress and cell death.

On the other hand, GSH does not react directly non-enzymatically with hydroperoxides. In fact, its role as a co-substrate for the selenium-dependent GPX has been recognized as the most important mechanism for reduction of H_2_O_2_ and lipid hydroperoxides. Moreover, more recently a family of proteins called peroxiredoxins has been recognized as catalyzing the reduction of H_2_O_2_ by GSH and/or other thiols, but with cysteine in its thiolate form, in their active sites rather than selenium-cysteine (Dickinson and Forman, 2002). GSH is also involved as an antioxidant in the detoxification of products deriving from ROS-promoted oxidation of lipids such as malonyl dialdehyde and 4-hydroxy-2-nonenal, and probably many other products of ROS interaction with cellular components (Comporti, 1987). The thiyl radicals formed from these reactions can also combine with different molecules, as well as with other thiyl radicals leading to the formation of GSSG in the latter instance. GSH forms also conjugates with a great variety of electrophilic compounds, when the electrophile is very reactive, or more often through the action of GST (Eaton and Bammler, 1999; Strange et al., 2000). Many other toxic metabolites are produced as side-products of the normal cellular metabolism and some of them can be also capable to react with GSH. For example, methylglyoxal, an enzymatic, and non-enzymatic product deriving from the glycolytic pathway (Martins et al., 2001; Inagi et al., 2010), is capable to interact with any molecules containing free amino groups such as amino acids, nucleotide bases, and cysteine residues in proteins (Li et al., 2008). Methylglyoxal and other *α*-dicarbonyls are also involved in ROS generation. GSH acts as a cofactor in the system of methylglyoxal elimination, which consists of two enzymes called glyoxalases (Yadav et al., 2008; Inagi et al., 2010). Other functions of GSH include: (i) maintaining the essential thiol status of cysteine residues on proteins; (ii) storage of cysteine reserves; (iii) involvement in the metabolism of estrogens, leukotrienes, and prostaglandins; (iv) participation in the production of deoxyribonucleotides; (v) participation in the maturation of iron–sulfur cluster in proteins; (vi) signal transduction from the environment to cellular transcription machinery (Dickinson and Forman, 2002).

## GSH AS MAIN REGULATOR OF CELLULAR REDOX STATUS AND REDOX SIGNAL TRANSDUCTION

The glutathione redox couple GSH/GSSG has a great importance in the cells and together with other redox-active couples, including NADPH/NADP^+^, Trx-SH/Trx-SS regulates and maintains the appropriate cellular redox status. The estimated *in vivo* redox potential for the GSH/GSSG couple ranges from-260 mV to –150 mV depending on the conditions (Jones, 2002). Thus, changes in the GSH/GSSG ratio are fundamental in the fine-tuning of signal transduction, even under mild oxidative stress that underlies physiological events such as cell cycle regulation and other cellular processes (Schafer and Buettner, 2001). Conditions characterized by increased ROS levels may require not only enhanced GSH action to maintain redox status, but also augmented energy supply and precursors to replace/enhance GSH content and/or transport it to the places where it is needed. However, when oxidative stress becomes prolonged and cellular systems are no more able to counteract the oxidative-mediated insults, the amount of free GSH decreases leading to irreversible cell degeneration and death.

Later on, additional roles for the antioxidant function of GSH have emerged that are strictly related to signal transduction: (i) the interaction of the tripeptide with NO or with RNS (see next section); (ii) the involvement of GSH in the process of protein *S*-glutathionylation. Protein *S*-glutathionylation is an important post-translation modification, providing protection of protein cysteines from irreversible oxidation and, at the same time, serving to transduce a redox signal by changing structure/function of the target protein. The process is observed either under massive increase of radical species or under physiological ROS flux. The process of *S*-glutathionylation may proceed spontaneously by thiol-disulphide exchange. However, for the majority of proteins these reactions could occur only under non-physiological GSH/GSSG ratio (i.e., 1:1). There is only the case of the nuclear transcription factor c-Jun that, due to an unusual redox potential, could react with GSSG at relatively high ratio of GSH/GSSG = 13 (Klatt et al., 1999). Therefore, it is likely that mechanisms of protein *S*-glutathionylation within the cells involves reaction of the “critical cysteine” on protein or GSH with a corresponding oxidized derivative such as S-nitrosyl (*S*-NO), sulfenic acid (*S*-OH), thiyl radical (*S*). Protein-sulfenic and glutathione sulfenic acids result from reaction with endogenously produced ROS or RNS but usually these species are rapidly transformed in prot-SSG (GSSR) or GSSG as more stable derivatives. Examples that a sulfenic acid intermediate is formed during redox regulation are become known for c-Jun, Fos, nuclear factor 1, nuclear factor-κB (NF-κB), GAPDH, and PTPs and up to date there are 2200 experimentally verified *S*-glutathionylated peptides from 169 research articles (Chen et al., 2014). The reverse reaction, de-glutathionylation, is mediated by the enzyme Grx. The cytosolic mammalian form, Grx1, operates via a nucleophilic ping–pong mechanism and is highly specific for *S*-glutathionylated proteins with respect to other mixed disulfides. Instead Grx2, a mitochondrial isoform, exhibits de-glutathionylation activity for peptides and proteins but with a catalytic activity 10-fold lower with respect to Grx1 (Beer et al., 2004; Grek et al., 2013). Moreover, the forward reaction of *S*-glutathionylation can be catalyzed by glutathione-*S*-transferase P (GSTP). At physiological pH, GSTP binds GSH and lowers the p*Ka* of the thiol, producing a thiolate anion (GS^-^) at the active site. This catalyzes the forward reaction, a specific example of which is provided by the reactivation of peroxiredoxin-6 (Prx). Indeed, it was demonstrated that heterodimerization of 1-cysPrx with GSH-saturated GSTP results in *S*-glutathionylation of the oxidized cysteine in 1-cysPrx followed by subsequent spontaneous reduction of the mixed disulfide and restoration of enzymatic activity (Manevich et al., 2004).

## GSH AND NITROSATIVE STRESS

Besides protecting against ROS, GSH is implicated in counteracting RNS-mediated damage. The intracellular GSH concentration appears to be an important factor in driving susceptibility to NO and its derivatives. NO is synthesized from L-arginine by enzymes known as nitric oxide synthases (NOSs; Knowles and Moncada, 1994). There are three genetically different isoforms of NOS. They include neuronal NOS (also known as nNOS, type I, NOS-1, and NOS-I), being the isoform found in neuronal tissues; inducible NOS (also known as iNOS, type II, NOS-II, and NOS2), being the isoform which can be synthesized following induction by pro-inflammatory cytokines or endotoxin and endothelial NOS (also known as eNOS, type III, NOS-III, and NOS-3), being the isoform expressed in endothelial cells (Murphy et al., 1993; Nelson et al., 2003; Maron and Michel, 2012).

Overproduction of NO can occur in several pathological conditions (e.g., inflammation, endotoxic shock, diabetes, ischemia/reperfusion injury; Wong and Billiar, 1995; Iadecola, 1997; Szabo, 1998; Friederich et al., 2009) or upon exposure to pharmacological drugs (Scatena et al., 2010) or ionizing radiation (Azzam et al., 2012). Overall these conditions stimulate the expression of iNOS, thus producing large amounts of NO. By a variety of mechanisms, NO can react with different molecules, under ambient oxygen, and the derived products can damage the cellular macromolecules, such as lipids, DNA bases, proteins as well as thiols. A primary reaction of RNS formation is the combination of NO and superoxide ($O_{2}^{•-}$) to form peroxynitrite (ONOO^-^) with a rate constant that is larger than that for the SOD-catalyzed dismutation of $O_{2}^{•-}$ (Jay-Gerin and Ferradini, 2000).

Following NO/ONOO^-^ exposure, significant effects on cellular GSH metabolism are reported. NO can react with the cysteine of GSH to form GSNO that may be considered an endogenous NO reservoir, which can release it when it reacts with Cu^+^ or with denitrosylating enzymes such as Trx and GSNO reductase (Sengupta and Holmgren, 2013). The same reaction can occur on protein cysteine, and the formed GSNO group functions as important intermediate in NO metabolism, a critical player in fulfilling the biolgogical function of NO in signal transduction (Chung, 2006). Besides functioning as a NO donor, GSNO can also mediate protein *S*-glutathionylation (Chiueh and Rauhala, 1999). GSNO can be also exported out of the cells via a the GSH transporter. In the extracellular milieu GSNO can undergo a transnitrosation reaction with cystine (Zeng et al., 2001) or thanks to catalytic activity of GGT generate S-nitrosocysteinylglycine that in turn can be cleaved by a membrane dipeptidase thus forming *S*-nitrosocysteine (S-NO). *S*-nitrosocysteine can enter the cells via the L-type amino-acid transporter (Hogg et al., 1997) and be reduced to release NO or transfer NO to proteins and thus mediate NO signaling. The way by which GSNO is formed has been the topic of several studies and many mechanisms have been proposed as extensively illustrated in the literature (Singh et al., 1996; Forman et al., 2004). Briefly, at typical physiological GSH concentrations and pH, the reaction between ONOO^-^ and GSH will predominantly be a two electron oxidation process leading to GSSG formation (Quijano et al., 1997). However, one electron oxidation process involving peroxynitrous acid and/or its derivatives can also occur. Under these circumstances a thiyl radical is formed that will initiate and propagate an oxygen-dependent chain reaction, involving peroxyl radical formation that will lead to further consumption of intracellular GSH (Quijano et al., 1997). Such reactions will subtract RNS from critical cellular targets, such as cell membrane lipids and proteins belonging to the complexes of the electron transport chain, thereby explaining why GSH status appears to be so critical in modulating the susceptibility to NO.

### GSH AS A PHYSIOLOGICAL MODULATOR OF NO HOMEOSTASIS

The importance of GSH in protecting the electron transport chain from ONOO^-^ is illustrated by experiments with GSH depleted astrocytes. Under such conditions marked damage to the ETC and cell death occurs following ONOO^-^ exposure (Barker et al., 1996). Conversely, the apparent increase in resistance of neurons to NO when co-cultured with astrocytes can be explained by considering the effect of co-culture upon the neuronal GSH concentration (Bolanos et al., 1996). Under these conditions, up-regulation of neuronal GSH is thought to occur as a result of the astrocytic release and preservation of GSH followed by cleavage, via GGT.

The effect of NO and its derivatives upon cellular GSH metabolism is also dependent on factors such as cell type, cellular environment and duration of exposure. For instance, induction of iNOS in microglia leads to a decrease in cellular GSH level, whereas, under comparable conditions, the GSH status of astrocytes remains largely unaffected (Bolanos et al., 1994; Chatterjee et al., 2000) Similarly, the GSH concentration in astrocytes does not appear to be affected by ONOO^-^ exposure (Bolanos et al., 1995). This apparent preservation of GSH may reflect the greater activity, in astrocytes, of GCL (Makar et al., 1994). Conversely, others and our laboratory have demonstrated that NO can favor GSH synthesis via the NEFL2-mediated transcription of GCL (Baldelli et al., 2008; Cortese-Krott et al., 2009).

The role of GSH as NO scavenger is also highlighted by Atakisi et al. (2010), who show how intraperitoneal administrations of GSH in rabbits cause a lowering of NO levels in plasma (Atakisi et al., 2010), suggesting that exogenous GSH may be a valuable enhancer of the antioxidant system. On the same line of evidence, supplementation of cells of neuronal origin with a membrane permeable GSH derivative (GSH60) protects them from NO neurotoxicity (Hu et al., 2012).

**FIGURE 1:**
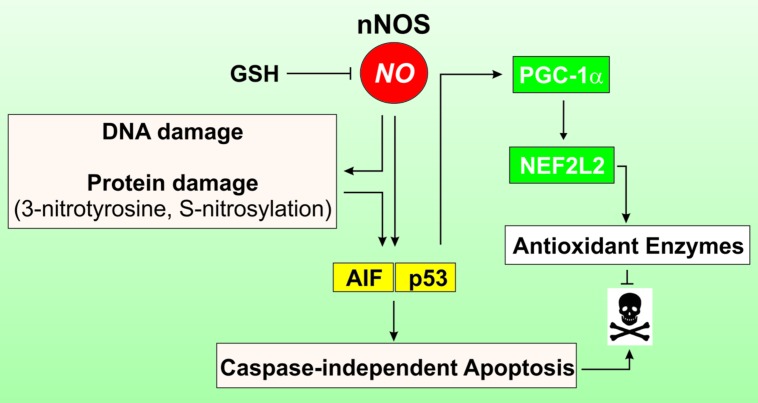
**Role of GSH in modulating cell response to NO.** In cells of neuronal or muscle origin NO is produced by neuronal nitric oxide synthase (nNOS). Intracellular GSH is a crucial factor in modulating NO reactivity, as it functions as an efficient NO buffer. When GSH levels decline, NO availability is increased and may trigger DNA damage as well as protein oxidation, in terms of *S*-nitrosylation of cyteines and formation of 3-nitrotyrosine on protein residues. This leads to induction of caspase-independent apotosis via the activation of the apoptosis inducing factor (AIF). Concomitantly, p53 is activated and binds to consensus sequence of PGC-1a promoter increasing its expression. PGC-1a in turn co-activates the NEFL2-dependent expression of antioxidant genes, thus limiting oxidative damage and cell death.

To investigate the importance of correct GSH homeostasis for the neurotrophic capacity of NO, Canals et al. (2001) pre-treated primary midbrain cultures with different doses of buthionine sulfoximine (BSO) a specific inhibitor of GCL, the rate-limiting enzyme in GSH synthesis. Under these conditions, NO triggers a programmed cell death with markers of both apoptosis and necrosis characterized by an early step of free radicals production followed by a late requirement for signaling on the sGC/cGMP/PKG pathway (Canals et al., 2001). Similarly, in our laboratory we demonstrated that neuronal cells are highly vulnerable to physiological flux of NO in the absence of GSH (Aquilano et al., 2011a). In particular, our study showed that the intracellular depletion of GSH is able to induce cellular stress in NO-producing cells through a NO-dependent mechanism, such as inhibition of CcOX activity, DNA damage, and S-NO and 3-nitrotyrosine (NO_2_-Tyr) protein accumulation (**Figure 1**). Moreover, NO seems to be the only mediator of cell proliferation arrest through the ERK1/2-p53 signaling pathway (Aquilano et al., 2011a). We also reported that even a slight and non-toxic decrease of GSH in brain mice causes protein nitration that is reversed by inhibiting NO production (Aquilano et al., 2011b). This evidence indicates that NO imbalance and the associated nitrosative stress observed in neruodegenerative disease and during aging are likely the consequence of the progressive decline of GSH (Aquilano et al., 2011b).

Next to this evidence, in our laboratory it has been demonstrated that GSH depletion modulates the peroxisome proliferator-activated receptor gamma, co-activator 1 alpha (PGC-1α) expression and its downstream metabolic pathway in neuronal and skeletal muscle cells (**Figure 1**). This effect was abrogated by inhibiting NOSs or guanylate cyclase, implicating NO/cGMP signaling pathway in this process (Aquilano et al., 2013). In particular, we found that the moderate depletion of GSH is operative upon fasting conditions in several organs including skeletal muscle and brain and this event is causative of the augmentation of NO availability. The increased NO bioavailability and the down-stream p53-mediated induction of PGC-1α favors the expression of NFE2L2 and its antioxidant-related genes (**Figure 1**). Thus, in these circumstances GSH, by subtracting biological active NO, could impede the activation of a more effective antioxidant response, which is involved in the increased lifespan.

## GSH TIPS THE SCALES BETWEEN SURVIVAL AND CELL DEATH

The ROS-mediated intrinsic pathway of apoptosis disclosed that the intracellular amount of GSH could determine the capability of the cell to undergo apoptosis. This process represents the summary of the GSH-related aspects with regards to: (i) changes in cellular GSH redox homeostasis through decreased GSH/GSSG ratio, due to either GSH oxidation or active GSH export in relation to the initiation or execution of the apoptotic cascade; (ii) evidence for *S*-glutathionylation in protein modulation and apoptotic initiation. The story about GSH and apoptosis starts around the 1990s with the evidence of an active GSH extrusion from the cell undergoing apoptosis (Ghibelli et al., 1995; van den Dobbelsteen et al., 1996) and with the subsequent conflicting notion that chemical depletion of GSH was not, however, sufficient to induce the apoptotic process (Ghibelli et al., 1999; Filomeni et al., 2005b). What is worth to summarize here is the fact that several pro-apoptotic stimuli induce an early extrusion of GSH from cells leading to a widespread mitochondrial damage and to cytochrome *c* release into the cytosol, the starting condition for apoptotic intrinsic pathway commitment. This event was, however, not necessarily preceded by an oxidative burst and later on we demonstrated that GSH release was the result of an active process that the cells carried out through specific carriers in order to efficiently execute the apoptotic program (Ghibelli et al., 1998). The picture illustrated was much more complex, because in several experimental systems, cell viability was not affected when GSH depletion was obtained chemically by inhibition of its synthesis. It was, therefore, concluded that GSH content is important for cell response to detrimental insults but not the principal event underlying the mitochondrial route of apoptosis. Finally, GSH depletion was designated as *necessary* to induce the redox unbalance characteristic of the induction phase of the mitochondrial pathway but not sufficient *per se* to assure the execution phase of apoptosis.

Successively the scenario was enriched by several molecular players that belong to redox-sensitive factors such as the Bcl-2 family (Hockenbery et al., 1993; Kowaltowski and Fiskum, 2005; Czabotar et al., 2014; Moldoveanu et al., 2014), p53 (Sun et al., 2003; Wang and Gu, 2014), c-Jun/AP-1 (Marshall et al., 2000), NF-κB (Pineda-Molina et al., 2001), heat shock factor 1 (HSF1; Jacquier-Sarlin and Polla, 1996; Anckar and Sistonen, 2011), and NE2FL2 (Itoh et al., 2004; Kansanen et al., 2013) together with the upstream mediators (dead receptors; Sullivan et al., 2000) of phosphorylative cascade, MAPKs (Filomeni et al., 2005c; Filomeni and Ciriolo, 2006), and downstream targets (mitochondrial and cytoskeleton proteins). Apoptosis began to be considered a much more fine-regulated process, being achieved only when the intracellular levels of GSH were affected in a well defined order, depending on the GSH-related redox pattern of the cell (Circu and Aw, 2012). The most intriguing and at the same time explanatory example comes from cancerous cells treated with ROS or with thiol-oxidizing agents. In particular, adenocarcinoma gastric (AGS) cells activated the mitochondrial pathway of apoptosis upon treatment with thiol-oxidizing agents, such as diamide, while they were resistant to hydrogen peroxide. Both responses correlated with GSH redox changes, with diamide increasing GSSG, and hydrogen peroxide inducing protein-GSH mixed disulfides (GSSR). We demonstrated that p53 was activated in response to diamide treatment by the oxidative induction of the Trx1/p38(MAPK) signaling pathway, which finally resulted in apoptosis. On the contrary, resistance to ROS was achieved by means of the redox activation of NE2FL2 (Filomeni et al., 2012). Neuroblastoma SH-SY5Y had a complete opposite behavior, being sensitive to hydrogen peroxide, but resistant to diamide. In this cells, the apoptotic pathway relied upon the same Trx1/p38MAPK/p53 signaling axis and cell survival to diamide relied upon redox activation of NE2FL2, in a way independent of Keap1 oxidation, but responsive to ERK1/2 activation (Filomeni et al., 2012). It was demonstrated that the molecular determinant(s) unifying these phenomena, was related to GSH and GSH-sensitive molecular factors. Indeed, SH-SY5Y cells showed high GSH levels but exhibited very low GPx activity, whereas AGS cells had low GSH content but several isoforms of the GPx enzyme.

The outstanding progress accomplished in the last years allows to assert that, even though the pathways implicated in the induction/execution of apoptosis have remained largely unaltered, many new redox-dependent processes have been added and we suggest the reading of several reviews on this subject (Anathy et al., 2012; Circu and Aw, 2012).

From the first evidence regarding the involvement of GSH in apoptosis, up to date many improvements have been made, and growing results, which attempted to dissect the mechanisms linking GSH to cellular redox-dependent processes, were added. The scenario depicted, clearly indicates that GSH is a *weighty protagonist* of the huge network governing the decision between life and death, through the modulation of cellular redox state. Indeed, GSH, Trx, Grx, Prx (together with the enzymes catalyzing their reduction), and the enzymatic antioxidant defense, as a whole, concur to modulate either ROS concentration, or the availability of cellular thiols, thus regulating the function of a large number of proteins implicated in the induction and/or execution of redox-sensitive pathways. Among such pathways ATG seems to be also included (Filomeni et al., 2010).

Autophagy is a degradation pathway essential for maintaining cellular homeostasis during different stressful conditions. Three distinct types of ATG are so far identified: (i) macro-ATG; (ii) micro-ATG, and (iii) chaperone-mediated ATG. Macro- ATG consists in the delivery of cytoplasmic cargo to the lysosome through the intermediary of a double membrane bound vesicle, the autophagosome, which fuses with the lysosome to form an autolysosome (Zhang, 2013). This process also contributes to the removal of damaged organelles, such as mitochondria and ER, and misfolded or aggregate-prone proteins (Mizushima, 2011). Moreover, as component of cellular integrated stress responses, ATG balances source of energy at critical times; e.g., under nutrient starvation, being able to provide substrates for ATP production and macromolecule synthesis (Zhang, 2013). Several ATG-related proteins were identified and their role in the molecular mechanisms of ATG highlighted. Since several ATGs and lysosomal cathepsin proteases depend on cysteines for their activities they can be the sensors or transducers of changes in the intracellular redox state through mechanisms involving redox signaling.

Accumulating data point to an essential role for ROS as mediators in the activation of ATG under several stimuli (starvation, pathogens, death receptors), even though the nature of this involvement remains still elusive. However, under starvation cells increase production of mitochondrial-derived hydrogen peroxide, which has been shown to be partly dependent on the activation of class III PI3K (Scherz-Shouval and Elazar, 2007). N-acetylcysteine, an efficient thiol antioxidant and GSH precursor, was able to inhibit ATG by impeding the lipidation process of LC3 mediated by ATG4, the activity of which is modulated by hydrogen peroxide (Scherz-Shouval et al., 2007). Successively, it was shown that TIGAR, a protein involved in the inhibition of the glycolytic pathway, could limit starvation-induced ATG by means of its ability to indirectly suppress ROS production (Bensaad et al., 2009). In fact, TIGAR redirects glucose-6-P toward the pentose phosphate pathway helping to lower ROS by the NADPH-dependent antioxidant systems. However, despite the plethora of observations regarding ROS and ATG, the only direct observation on the involvement of signal transduction mechanisms regulating self-digestion upon thiol/disulfide changes was evidenced in yeasts (Deffieu et al., 2009). It was demonstrated that GSH level affects the selective elimination of mitochondria (mitophagy) in *S. cerevisiae.* Also in this case the thiol antioxidant *N*-acetyl-cysteine prevented the delivery of mitochondria to the digestive vacuoles. The inhibition was specific for mitophagy because neither macro- nor micro-ATG was altered. Moreover, chemical or genetic manipulation of GSH pool stimulated mitophagy but not general ATG. These data, while revealing that mitophagy can be regulated independently of general ATG, at the same time, outlined a role for cellular redox status in its commitment.

**FIGURE 2:**
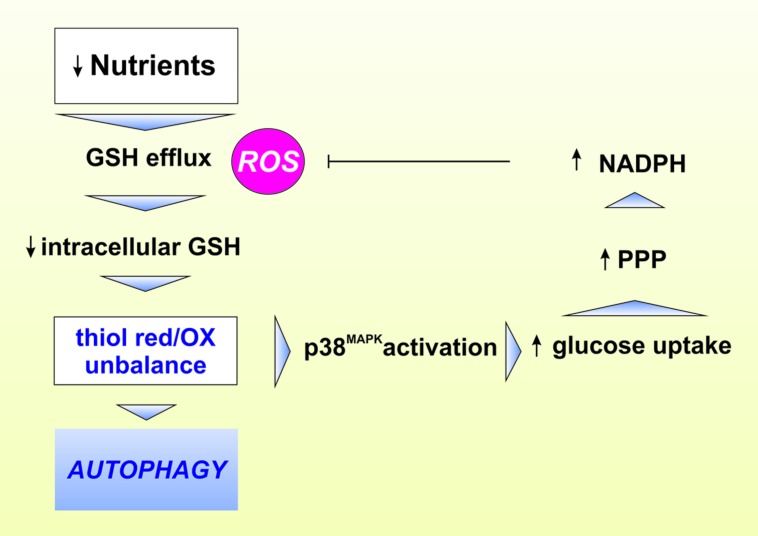
**Role of GSH in the modulation of ATG.** Lack of nutrients leads to increased reactive oxygen species (ROS) production concomitant to intracellular GSH eﬄux through the cell membrane. These events result in thiol redox unbalance leading to activation of crucial proteins involved in ATG induction and execution. Under nutrient restriction, the phoshorylative signaling cascade governed by p38^MAPK^ favors glucose uptake leading to the increase of NADPH production via the enhancement of pentose phosphate pathway (PPP). NADPH in turn contributes to buffer higher ROS-mediated oxidative damage.

These observations allowed postulating that intracellular redox state of the thiol pool, which strongly depends on GSH level, can drive autophagic response at multiple levels (Filomeni et al., 2010). We demonstrated that, although ROS are necessary for the initiation phase of ATG, the modulation of thiols are fundamental for the progression of starvation-induced ATG (Desideri et al., 2012; **Figure 2**). Indeed, upon nutrient deprivation GSH was efficiently extruded from cells. This process, along with GCL inhibition and the formation of mixed disulfides between GSH and protein sulfhydryl, concurred to a steady-state decrement of free GSH concentration and, consequently, to an intracellular redox state shift toward more oxidizing conditions, which could be required for avoiding fast reduction of oxidized cysteines allowing the upstream ROS-induced autophagic stimulus to be propagated. From these results it is emerging that, under increased ROS flux, the cell necessitates to efficiently extrude GSH in order to propagate the redox stimulus. Moreover, ATG and apoptosis induction had common starting condition with ATG underlying first cell response (survival) before commitment to apoptosis. More recently, we also evidenced that starvation induced p38^MAPK^ activation, which by reprogramming glucose metabolism by the pentose phosphate pathway sustains NADPH production. This process was fundamental in order to buffer ROS flux and a consequent detrimental over increase of self-digestion (Desideri et al., 2014; **Figure 2**). Overall the above reported evidence on the involvement of GSH and the redox environment are only some issues that deserve to be further investigated to comprehend the several aspects characterizing the possible crosstalk between redox reactions and modulation of ATG. Indeed, redox modulation of ATG could impinge several fields interconnected with bioenergetics and metabolic adaptations to different stimuli. In this context, the peptidase, DJ-1 could have a prominent role because it possess reactive cysteines and is a modulator of mitochondrial dynamics and mitophagy (Thomas et al., 2011). Moreover, recently it has been reported that the pathway governed by NE2FL2 is not only regulated by the proteasome but also by ATG (Jain et al., 2013). The p62/SQSTM1 (sequestosome 1) protein, which acts as a cargo receptor for autophagic degradation of ubiquitinated proteins, is up-regulated by various stressors. The inhibitory subunit of NE2FL2, Keap1, is a cysteine-rich protein that serves as a redox sensor and can bind to p62 and be degraded by autophagic activity. In response to oxidants, cysteine modification on Keap1 releases NE2FL2, which is then stabilized and transported to the nucleus where it activates transcription of antioxidant genes, including proteasomal PA28αβ. This results in increased proteasomal degradation of oxidatively damaged proteins as well as p62, thereby facilitating ATG-lysosomal degradation of proteins and dysfunctional organelles, such as the mitochondria (Levonen et al., 2014).

Apoptosis and ATG are processes altered in cancer cells that show critical metabolic transformations associated with enhanced cellular stress. Adaptation to such conditions is characteristic of cancer cells survival and the underlying processes of resistance to apoptosis and/or efficient ATG induction. Therefore, antioxidant therapy was acclaimed as a valuable tool to selectively kill cancer cells. Results obtained from randomized clinical trials, however, were inconsistent and in some circumstances have indicated that antioxidants increase cancer risk (Klein et al., 2011; Watson, 2013). Moreover, it was reported that supplementing the diet with the antioxidants *N*-acetylcysteine and vitamin E markedly increases tumor progression and reduces survival in mouse models of B-RAF- and K-RAS-induced lung cancer, by reduction of ROS, DNA damage, and p53 expression (Sayin et al., 2014). On the contrary, the use of small molecules derived from diet that alter the levels of ROS, such as diallyl disulfide, polyphenols, isothiocyanates, and terpenoids has been suggested for the treatment of cancer by promoting ROS generation and GSH depletion in cancer cells (Filomeni et al., 2003; Shankar et al., 2006; Trachootham et al., 2006; Yue et al., 2006; Aquilano et al., 2010). Interestingly, depletion of mitochondrial GSH has also been associated with apoptosis or ATG induced by chemotherapeutic drugs (Samudio et al., 2005; Chen et al., 2011).

From these data we can reasoned that by selectively targeting GSH content of cancer cells we can induce apoptosis either directly or by combining therapies with redox antineoplastic agents.

## GSH AND VIRAL INFECTION

In this section we plan to discuss the role of the tripeptide GSH in the process dealing with modulation of viral infection from the point of view of intracellular redox shift necessary for the efficient replication cycle. We are aware that several other microbial organisms alter the GSH content of cell and infected organism and very recently it was published a review on GSH and infection (Morris et al., 2013) that we suggest for readers who are interested in more wide knowledge on this topic. The choice is also dictated by the limit in length imposes for this review.

One of the first evidence involving GSH homeostasis alteration in viral infection was a paper published by Buhl et al. (1989). In this study, a 30% decrease in venous plasma and 60% decrease in epithelial lung fluid of asymptomatic HIV-seropositive subject were registered. Since GSH is a powerful stimulator of immune function, it was hypothesized that its loss could be critical for the immunodeficiency onset during HIV infection. Successively, oxidative phenomena and GSH were involved in the induction of HIV expression. Indeed, the supplementation of GSH precursors N-acetyl cysteine or GSH esther to HIV-infected cultured monocytes was found to efficiently inhibit HIV expression (Kalebic et al., 1991). Then, other reports suggested that impaired antioxidant defense, and in particular altered GSH metabolism, play important roles in HIV infection. Indeed, HIV-infected cells, besides having decreased level of intracellular GSH, display increased generation of ROS along with high rate of GSSG formation (Droge et al., 1994). T cells isolated from HIV-infected patients had lower cysteine and GSH contents (de Quay et al., 1992; Staal et al., 1992). Moreover, GSH levels were found decreased in peripheral blood mononuclear cells, and monocytes of HIV infected individuals (Buhl et al., 1989; Morris et al., 2013). Notably clinical studies carried out in 1997 directly demonstrated that low GSH levels in CD4 T cells predict poor survival in otherwise indistinguishable HIV-infected subjects (Herzenberg et al., 1997). Importantly, the oral administration of *N*-acetyl cysteine restored GSH blood levels, suggesting the use of this drug for HIV treatment and improving survival. Overall these findings point that the reducing milieu created by GSH inhibits the triggering of oxidation-dependent signal transduction pathways associated with HIV expression. Actually, decreased GSH levels are known to activate NF-κB transcription factor, which has been shown to bind and activate genes controlled by the HIV long terminal repeat (Staal, 1998), and thus may affect viral replication.

**FIGURE 3:**
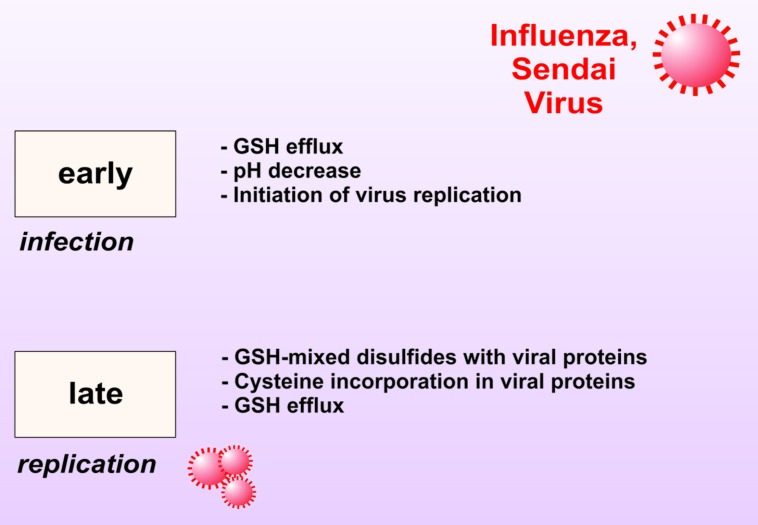
**Modulation of intracellular GSH level during Sendai and Influenza virus infection.** In the early phase of infection, host cell membrane perturbation causes decrease of intracellular pH and GSH eﬄux allowing virus cycle start. In the late phase of infection, GSH is also engaged in forming mixed disulfides with viral proteins. GSH intracellular levels are further decreased as its component cysteine is incorporated into viral proteins.

Successively, GSH was also found to be a powerful inhibitor of other viruses including herpes simplex virus type 1 (HSV-1), Sendai and influenza. We have demonstrated that during viral infections a decrease of intracellular GSH is operative that varies in intensity, duration and mechanism of induction depending on the type of virus and the infected host cell (**Figure 3**; Garaci et al., 1992, 1997; Palamara et al., 1995, 1996; Ciriolo et al., 1997). We demonstrated that GSH supplementation directly inhibits the production of mature viral particles by interfering with virus envelope glycoproteins. Indeed, administration of GSH permeable analog GSH-C4 blocks the replication of several influenza virus strains (Sgarbanti et al., 2011). All viral glycoproteins share the characteristic of their assembly into oligomers that necessitates the formation of a disulfide bond and this is inhibited by the reducing action of GSH. For instance, the influenza HA glycoprotein is a homotrimer in which each monomer consists in two-disulfide linked subunits. Cells harboring high levels of GSH efficiently counteract the glycoprotein assembly and coherently the intracellular depletion of GSH, by its synthesis inhibitor BSO, favors influenza virus replication (Nencioni et al., 2003). To set a more suitable pro-oxidant milieu critical for maturation of HA, influenza virus also impinges GSH loss (Nencioni et al., 2003; **Figure 3**). Moreover, we also found that a strict relationship exists between the level of the GSH intracellular content, anti-apoptotic protein Bcl-2 and the intensity of influenza virus replication. In particular, we substantiated that high levels of Bcl-2 leads to increased amount of GSH and finally impair virus infection (Nencioni et al., 2003). However, Bcl-2 and GSH interfere with two independent routes of viral replication cycle. Indeed, while GSH negatively impact with the expression of late viral proteins (in particular hemagglutinin and matrix), Bcl-2 impairs nuclear-cytoplasmic translocation of viral ribonucleoproteins (vRNPs). In this regard, we have successively documented that the formation of a Bcl-2/p38MAPK heterocomplex into the cytoplasm is the genuine responsible for the inhibition of vRNPs nuclear export. More in dept, Bcl-2 impedes p38MAPK translocation into the nucleus, wherein it should be responsible for phosphorylation-dependent NP export from the nucleus. As result, the blockage of NP into the nucleus dramatically affects viral packaging and final maturation (Nencioni et al., 2009).

Also other authors have shown the importance of GSH for influenza virus infection and replication. In fact, Madin-Darby canine kidney cells or human small airway epithelial cells infected with influenza virus and treated with reduced GSH exhibited an inhibition of viral matrix proteins. Moreover in BALB/c mice, inclusion of GSH in the drinking water decreased viral titer in both lung and trachea homogenates after intranasal inoculation with a mouse-adapted influenza strain A/X-31 (Cai et al., 2003).

Our group demonstrated that GSH was able to inhibit also HIV expression in human macrophages at late stages and this was related to the selective decrease of specific glycoproteins, such as gp120, which are notably rich in disulfide bonds (Garaci et al., 1997).

Next to this, it has been also reported that *in vitro* infection and replication of human herpes simplex virus type 1 (HSV-1) induced a dramatic decrease in intracellular GSH. The addition of exogenous GSH was not only able to restore its intracellular levels almost up to those found in uninfected cells, but also to completely inhibit the HSV-1 replication (Palamara et al., 1995). Similarly, it has been reported that Madin-Darby canine kidney cells infected with Sendai virus rapidly lost GSH, without increase in the oxidized products. In this condition of viral infection, oxidative stress is imposed by GSH depletion, occurring in two steps and following direct virus challenge of the cell membrane, without the intervention of ROS (Ciriolo et al., 1997). Furthermore, we have demonstrated that a direct administration of high doses of GSH exerts antiviral activity and improves immune functions in a murine immunodeficiency animal model. In fact, evaluation of pro-viral DNA content showed that GSH was effective in inhibiting the infectivity of murine leukemia virus-infected mice (LP-BM5) in lymph nodes, spleen, and bone marrow. Thus, GSH reduces the pro-viral DNA load in the first period of infection, suggesting that this antioxidant may be useful for improving current antiviral therapies (Palamara et al., 1996).

Also the pathogenesis of dengue virus (DV) infection has been closely linked to the GSH concentration. In fact, Tian et al. (2010) have demonstrated that DV serotype 2 (DV2) infection resulted in a decrease in intracellular GSH level, which caused NF-κB activation and increased DV2 production (Tian et al., 2010). Supplemental GSH significantly inhibited activation of NF-κB, resulting in a decreased production of DV2 in HepG2 cells. Furthermore, high activity of NF-κB and increased production of DV2 was observed in HepG2 cells treated with BSO. In conclusion, DV2 infection could reduce host intracellular GSH concentration and benefited from this process. Supplemental GSH could inhibit viral production, indicating that GSH might be valuable in the prevention and treatment of DV2 infection (Tian et al., 2010). Additionally, it has been demonstrated that also chronic hepatitis C virus (HCV) infection in hepatocyte cell lines led to a decreased levels of reduced GSH and a concomitant increase of oxidative stress (Abdalla et al., 2005).

The altered intracellular redox state has been envisaged as target for anti-influenza therapy, but also in this case the data arising from *in vivo* studies are inconsistent, and in some cases have indicated that antioxidants could exacerbate the disease. The most ascertain example is the use of vitamin C in the prevention and treatment of the common cold, which has been a subject of controversy for at least 70 years (Hemila and Chalker, 2013). However, Friel and Lederman (2006) published that a nutritional supplementation of antioxidants may be effective against the influenza A (H5N1) infection. The cocktail suggested included also GSH and its precursor *N*-acetylcysteine that could avoid the rapid and detrimental loss of the intracellular content of the tripeptide during infection. In this case, as also stated by the authors, the supplementation could be efficacious only if it is going to be prophylactically used prior to an H5N1 influenza infection. However, no data are available on the real efficacy of such formulation on the pathogenesis of H5N1 influenza.

## CONCLUSION

Glutathione displays remarkable metabolic and regulatory versatility, which poses the tripeptide at the center stage of a multitude of cellular processes, including cell proliferation, differentiation, and death. Some of the molecular mechanisms underlying the modulation of such processes by GSH have been established and mainly linked to the modulation of cellular redox state. The most intriguing aspect that is nowadays emerging is the crosstalk between ATG and apoptosis through a link with redox signaling governed by GSH. We expect in the near future growing recognition of GSH involvement, by *S*-glutathionylation and/or by the thiols redox state shift, not only in the induction of ATG and/or apoptosis, but also in the modulation of activities of molecular/transcription factors such as NEF2L2, PGC1α, and p53 that by inducing the antioxidant defense and assuring mitochondrial homeostasis could block both processes.

Finally, GSH deficiency contributes to oxidative/nitrosative stress, a condition actually connected with the pathogenesis of many diseases, including cancer, diseases of aging, cystic fibrosis, infection and neurodegeneration. Therefore, elucidating the mechanisms through which GSH is involved in ATG and/or apoptosis will be crucial to develop advanced therapies to counteract or ameliorate such diseases.

## Conflict of Interest Statement

The authors declare that the research was conducted in the absence of any commercial or financial relationships that could be construed as a potential conflict of interest.
